# A Multi-Scale Vision–Sensor Collaborative Framework for Small-Target Insect Pest Management

**DOI:** 10.3390/insects17030281

**Published:** 2026-03-04

**Authors:** Chongyu Wang, Yicheng Chen, Shangshan Chen, Ranran Chen, Ziqi Xia, Ruoyu Hu, Yihong Song

**Affiliations:** 1 China Agricultural University, Beijing 100083, China; 2 National School of Development, Peking University, Beijing 100871, China

**Keywords:** insect pest management, small-target pest recognition, intelligent plant protection, precision agriculture monitoring, deep learning for insect identification

## Abstract

In complex agricultural production environments, small-target pests—characterized by tiny scales, strong background confusion, and close dependence on environmental conditions—pose major challenges to precise monitoring and green pest control. To facilitate the transition from experience-driven to data-driven pest management, a multi-scale vision–sensor collaborative recognition method is proposed for field and protected agriculture scenarios to improve the accuracy and stability of small-target pest recognition under complex conditions. The method jointly models multi-scale visual representations and pest ecological mechanisms: a multi-scale visual feature module enhances fine-grained texture and morphological cues of small targets in deep networks, alleviating feature sparsity and scale mismatch, while environmental sensor data, including temperature, humidity, and illumination, are introduced as priors to modulate visual features and explicitly incorporate ecological constraints into the discrimination process. Stable multimodal fusion and pest category prediction are then achieved through a vision–sensor collaborative discrimination module. Experiments on a multimodal dataset collected from real farmland and greenhouse environments in Linhe District, Bayannur City, Inner Mongolia, demonstrate that the proposed method achieves approximately 93.1% accuracy, 92.0% precision, 91.2% recall, and a 91.6% $F_{1}$-score on the test set, significantly outperforming traditional machine learning approaches, single-scale deep learning models, and multi-scale vision baselines without environmental priors. Category-level evaluations show balanced performance across multiple small-target pests, including aphids, thrips, whiteflies, leafhoppers, spider mites, and leaf beetles, while ablation studies confirm the critical contributions of multi-scale visual modeling, environmental prior modulation, and vision–sensor collaborative discrimination.

## Simple Summary

Small-target insect pests pose a major challenge to intelligent agricultural monitoring due to their tiny size, complex backgrounds, and strong dependence on environmental conditions. To address these issues, this study proposes a multi-scale vision–sensor collaborative framework that integrates visual imagery with environmental sensing data for accurate pest recognition. The model first captures fine-grained pest features through multi-scale visual representation learning, and then introduces environmental factors—such as temperature, humidity, and illumination—as prior information to guide feature discrimination. A collaborative fusion mechanism is further designed to enhance cross-modal consistency and improve classification robustness. Experiments conducted on a real multimodal pest dataset collected from farmland and greenhouse environments demonstrate that the proposed method achieves 93.1% accuracy, 92.0% precision, 91.2% recall, and a 91.6% F1-score, outperforming conventional machine learning and single-modality deep learning approaches. These results indicate that integrating ecological sensing with computer vision can provide a reliable technical pathway for early pest detection and precision agricultural management.

## 1. Introduction

Accurate and early identification of agricultural pests is a core part of crop protection and green pest management, which is critical to protecting crop yield and quality, cutting chemical pesticide use and preserving agricultural ecological security [1]. In the early crop growth stage, pest populations are small and damage symptoms are inconspicuous, so timely monitoring is essential for precise intervention without large-scale pesticide application, thus reducing control costs and ecological risks [2]. Small-scale pests like aphids and thrips are particularly challenging to identify due to their tiny size, rapid reproduction, dense aggregation and cryptic habitats on leaf undersides or in plant gaps, which cause their populations to expand quickly and threaten crop growth persistently, making them a key difficulty in intelligent field monitoring and automated recognition [3]. With the advancement of high-resolution imaging, intelligent trapping systems and agricultural IoT technologies, computer vision-based pest recognition has become a vital research direction in smart agriculture, enabling all-weather, low-cost and large-scale monitoring [4].

Before deep learning was widely adopted, pest identification mainly depended on traditional machine vision combined with expert knowledge [5], using handcrafted color, texture or morphological features with threshold segmentation, edge detection or shallow classifiers [6]. However, the complex leaf textures, drastic illumination changes and subtle morphological differences of small pests in real field environments make these methods highly sensitive to imaging conditions and poor in generalization [7]. Additionally, handcrafted features fail to capture the diverse morphology and spatial distribution of pests, leading to frequent false and missed detections in complex backgrounds [8], which greatly restricts the application of traditional methods in small-target pest recognition [7]. With the rapid development of deep learning [9], convolutional neural networks (CNNs) and their variants have shown strong feature learning capabilities in agricultural pest recognition [10]. End-to-end training enables the automatic learning of discriminative features, partially overcoming the limitations of manual feature design [11], and attention mechanisms and Transformer architectures further boost global contextual modeling [12]. Nevertheless, most existing deep learning-based methods rely on a single visual modality, and their performance in small-target pest recognition is still limited under complex field conditions [13]. Small targets occupy only a tiny proportion of image pixels, and their features are easily weakened or lost during repeated downsampling [14]; meanwhile, illumination variation, occlusion, background noise and imaging blur further intensify target–background confusion [15]. More importantly, the intrinsic ecological relationships between pest occurrence and environmental conditions are often neglected, and environmental factors such as temperature, humidity and illumination are rarely used as prior constraints [16].

Recent studies have explored advanced visual modeling strategies: Lyu et al. [17] proposed a CNN–Transformer hybrid framework for multi-label natural enemy–pest detection, achieving remarkable improvements in F1-score and mAP; Zhang et al. [18] introduced the DFRCP framework to enhance pest damage detection in motion-blurred images, with a performance gain of about 10.4% when combined with YOLOv11; Vhatkar et al. [19] proposed an optimization-assisted SSD integrated with graph attention networks to improve pest detection in complex backgrounds; and Seol et al. [20] enhanced spray perception accuracy in precision aerial spraying via spatial–temporal filtering and 3D deep learning. Despite these progress, automatic recognition of small-target pests still faces three key challenges: the scale mismatch between small targets and deep network receptive fields, high target–background similarity under complex conditions, and inadequate modeling and fusion of environmental prior information. To tackle these challenges, a multi-scale vision–sensor collaborative pest recognition framework is proposed. Multi-scale visual feature modeling strengthens the fine-grained representation of small targets. From a biological perspective, instantaneous microclimate conditions—such as immediate temperature, humidity, and illumination at the exact time of photography—heavily influence the acute behavioral responses of small arthropods, such as their tendency to seek shade on leaf undersides or within plant crevices during intense midday heat and light. By capturing these acute behavioral contexts, instantaneous environmental sensor data is introduced as prior modulation signals to guide the model to generate ecologically plausible visual responses, thereby improving recognition performance in complex agricultural scenarios. The main contributions of this study are formulated as follows.

1. A multi-scale visual feature modeling mechanism for small-target pests is proposed, where cross-scale information fusion effectively mitigates feature sparsity and scale mismatch problems;
2. A vision–sensor collaborative prior modulation strategy is designed to deeply integrate instantaneous environmental sensor information into the pest recognition process, enhancing the model’s robustness under complex local conditions;
3. Systematic experiments on real field-collected datasets verify that the proposed method significantly outperforms multiple mainstream approaches in small-target pest recognition tasks;
4. The proposed framework enables early and accurate identification of small-target pests to support precision pest management, which is potentially beneficial for reducing pesticide input, lowering production costs, and improving agricultural productivity.

## 2. Related Work

### 2.1. Deep Learning-Based Pest Recognition Methods

With the growing adoption of computer vision and artificial intelligence in agriculture, automatic pest recognition has evolved from traditional machine vision techniques to deep learning-based models [21]. Early approaches relied on handcrafted color, texture, and morphological features combined with shallow classifiers such as support vector machines and random forests [22]. However, these methods are highly dependent on feature engineering and imaging conditions, making them sensitive to illumination variation, background complexity, and individual morphological differences [23]. In real field environments, complex leaf textures and background noise, together with the high visual similarity between small pests and surrounding backgrounds, significantly limit the generalization ability and stability of traditional methods [24]. With the emergence of convolutional neural networks, deep learning-based pest recognition has become the dominant research paradigm [25]. CNNs can automatically learn hierarchical feature representations through multi-layer convolution and nonlinear mappings, partially overcoming the limitations of manual feature design [26]. Classical classification networks and detection frameworks have been introduced into pest recognition and have achieved promising results under controlled conditions or relatively simple backgrounds [27]. Attention mechanisms and Transformer architectures have further been explored to enhance global contextual modeling and mitigate background interference [28]. Nevertheless, most existing models are originally designed for medium- and large-scale objects, and their downsampling and feature extraction strategies are not tailored to small-target pests [29]. When targets occupy only a small fraction of image pixels, fine-grained details are easily weakened or lost during deep feature extraction, resulting in limited discriminative capability for pests such as aphids and thrips [30]. This limitation becomes more pronounced in complex field environments and remains a key bottleneck in current deep learning-based pest recognition research [31].

### 2.2. Small-Target Visual Recognition and Multi-Scale Modeling

To address the challenges of small-target recognition, multi-scale modeling has become an important research direction in computer vision [32]. The core idea is to jointly model target features across multiple scale spaces to compensate for information loss at a single scale [33]. Representative approaches include feature pyramid networks, multi-level feature fusion architectures, and cross-scale attention mechanisms [34]. By integrating high-resolution shallow features with low-resolution semantic features, these methods have substantially improved small-target detection performance in general object detection tasks [35]. Theoretically, multi-scale modeling helps alleviate scale mismatch by preserving spatial details while introducing semantic abstraction [36]. However, most existing multi-scale methods are designed for structured scenarios such as urban environments or remote sensing imagery, where background patterns are relatively regular [37]. Agricultural scenes are inherently unstructured, characterized by complex crop morphologies, repetitive textures, and significant appearance variations of pests across growth stages and environmental conditions [38]. When directly applied to pest recognition tasks, existing multi-scale methods often fail to fully exploit their advantages, and problems such as target–background confusion and high false-detection rates persist [39]. Consequently, designing multi-scale visual modeling strategies that are better adapted to small-target pests in agricultural scenes remains an open research problem [40].

### 2.3. Multimodal and Sensor-Assisted Agricultural Perception Methods

Pest occurrence processes are closely related to environmental conditions, providing a theoretical basis for multimodal and sensor-assisted agricultural perception [41]. Numerous studies have shown that factors such as temperature, humidity, and illumination significantly influence pest growth, activity patterns, and population dynamics. Accordingly, environmental sensors have been widely deployed for pest outbreak analysis and early risk warning [42]. Most existing studies model sensor data as time-series signals using statistical or machine learning approaches, offering macro-level decision support for agricultural management. However, sensor information is typically decoupled from visual recognition, limiting its contribution to fine-grained perception tasks [43]. From a deep learning perspective, current multimodal agricultural perception methods remain limited in collaborative modeling between visual and sensor information [44]. Some studies simply concatenate environmental variables with visual features as additional inputs [45], but such shallow fusion strategies fail to fully exploit the guiding role of environmental information in visual discrimination [46]. This limitation is particularly evident in small-target pest recognition, where performance gains are often unstable [47]. Moreover, environmental conditions are rarely treated as ecological priors to modulate visual feature learning, causing models to rely predominantly on visual cues and making robust recognition under complex field environments difficult to achieve [48].

## 3. Materials and Method

### 3.1. Data Collection

The dataset used in this study was collected from representative open-field and facility-based agricultural production environments in Linhe District, Bayannur City, Inner Mongolia. It should be noted that all field data originate from the same geographic region. Although this ensures environmental consistency and controlled ecological variation, it may limit the direct generalization of the model to regions with substantially different climatic conditions, cropping systems, or pest population structures. This region is located in the Hetao Irrigation Area, where agricultural cultivation is intensive and pest occurrence exhibits strong seasonality and representativeness. In addition to field-collected data, a small portion of supplementary data was obtained from publicly available online sources. The data collection period spanned from April 2023 to November 2023, covering multiple critical crop growth stages, including the seedling stage, vegetative growth stage, and early fruiting stage, while also encompassing typical climatic conditions such as spring–summer transition, high-temperature drought, and large diurnal temperature variations. Pest image data were mainly acquired from open-field vegetable plots and solar greenhouse environments, with crop types including maize, tomato, and pepper. These settings effectively reflect the real-world distribution characteristics of pests in local agricultural production. The dataset consists of both image data and environmental sensor data, as shown in Table 1.

**Figure 1:**
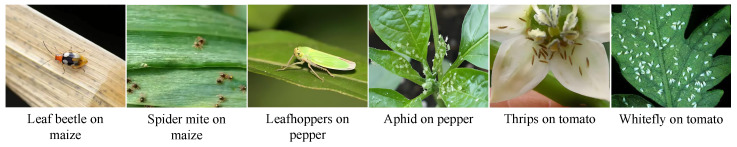
Instances of crops and common insect pests encompassed within the dataset.

Image data were acquired using a combination of fixed agricultural monitoring cameras and handheld high-definition cameras. Fixed cameras were deployed between crop rows or at the top of greenhouses to continuously record pest occurrence and activity under natural conditions, whereas handheld cameras were used to supplement multi-view and local high-resolution images in order to enhance the visibility of small-target pests. During acquisition, original image resolutions were preserved as much as possible, with resolutions mainly ranging from 1920×1080 to 3840×2160, so as to avoid the loss of fine-grained details of small targets caused by excessive compression. All images were collected under natural illumination without artificial intervention in the field environment, thereby ensuring the authenticity and complexity of the data. In terms of pest categories, the dataset focuses on small-target pests that are common in Linhe District and difficult to control. Specifically, the monitored arthropods and their corresponding host crops include the green peach aphid (*Myzus persicae*) and leafhoppers (*Empoasca* spp.) infesting peppers, western flower thrips (*Frankliniella occidentalis*) and sweetpotato whiteflies (*Bemisia tabaci*) on tomatoes, as well as two-spotted spider mites (*Tetranychus urticae*) and leaf beetles (*Monolepta hieroglyphica*) on maize, as shown in Figure 1. The density levels of these arthropods varied significantly across the captured data, ranging from low-density occurrences with a few scattered individuals to high-density colonies characterized by severe spatial overlap and aggregation. These pests are generally small in size and tend to aggregate on the undersides of leaves, young shoots, or near leaf veins. Under conditions of complex leaf textures and varying illumination, they are easily confused with background regions, exhibiting typical characteristics of small-target recognition tasks. All image samples were annotated frame by frame by professionals with plant protection expertise, followed by cross-validation procedures to ensure the accuracy of pest categories and annotated regions.

In addition to visual images, environmental sensor data temporally aligned with the image data were synchronously collected to characterize the ecological background of pest occurrence. Sensor nodes were deployed within the data collection areas to continuously record key environmental variables, including air temperature, relative humidity, and illumination intensity. The sampling frequency was set to once every 10 min, and precise temporal alignment with image data was achieved using timestamps. During data acquisition, anomaly monitoring was conducted in real time, and missing values were subsequently corrected and normalized during post-processing.

Image data were collected using fixed agricultural monitoring cameras (Hikvision DS-2CD3T47G2-L, Hikvision, Hangzhou, China) and handheld high-definition cameras (Canon EOS 90D, Canon Inc., Tokyo, Japan) equipped with EF-S 18–135 mm lenses. During acquisition, exposure time was adaptively set within the range of 1/60–1/200 s under natural illumination, and white balance was configured in automatic mode to accommodate dynamic field lighting conditions. Environmental variables were synchronously recorded using integrated agricultural sensor nodes (Model: RS-WS-N01, Renke Instruments, Yantai, Shandong, China). Air temperature was measured in degrees Celsius (°C), humidity was defined as relative humidity (%RH), and illumination intensity was measured in lux. All sensors were calibrated prior to deployment to ensure measurement consistency across different acquisition sites.

### 3.2. Data Pre-Processing and Augmentation Strategy

In complex agricultural scenarios, small-target pest recognition tasks are often affected by issues such as inconsistent image resolutions, significant background noise, extremely small-target scales, and temporal asynchrony among multimodal data sources. If these factors are not properly addressed, the convergence stability and generalization performance of deep models are directly degraded. Therefore, systematic pre-processing and augmentation of both image data and environmental sensor data before model training constitute a critical prerequisite for ensuring the effectiveness of the proposed multi-scale vision–sensor collaborative framework.

For visual data, differences in acquisition devices and capture distances typically lead to large variations in image resolution, whereas deep learning models generally require fixed input sizes to enable batch training and feature alignment. Based on this principle, resolution normalization is first applied to all raw images. Let a raw image be represented as $I\in R^{H\times W\times C}$, where *H*, *W*, and *C* denote the image height, width, and number of channels, respectively. Through bilinear interpolation, the image is mapped to a unified resolution $H_{0}\times W_{0}$, which can be expressed as

(1) $$I^{\prime }=R\left(I;H_{0},W_{0}\right),$$

where R(·) denotes the resolution resampling operator. This process preserves the overall structural information while ensuring spatial-scale comparability across images from different sources, thereby providing a unified input basis for subsequent multi-scale feature modeling. However, simple global resizing may further reduce the pixel proportion of small-target pests, which weakens their discriminative features. To alleviate this issue, a cropping-based local enhancement strategy is introduced on top of resolution normalization. Local regions are randomly selected from each image for cropping and then rescaled to the original input size, so that small targets occupy a larger relative proportion in the cropped images. Let the top-left coordinate of the cropping region be (x,y), with cropping width $w_{c}$ and height $h_{c}$. The cropping operation is formulated as

(2) $$I_{c}=I^{\prime }\left(x:x+w_{c},;y:y+h_{c}\right),$$

after which a scale transformation is applied to map $I_{c}$ back to the unified input resolution. In this manner, the relative scale of small targets is increased without modifying the network architecture, thereby mitigating the progressive weakening of small-target features in deep networks from the data perspective. On this basis, a data augmentation mechanism combining random scale perturbation and local magnification is further designed for small-target pests. The core idea is to impose random perturbations on target scales during training to enhance the model’s adaptability to scale variations. Specifically, a random scaling factor α is applied to the cropped image $I_{c}$, where α follows a uniform distribution over the interval $\left[\alpha_{\min},\alpha_{\max}\right]$, i.e.,

(3) $$\alpha ∼U(\alpha_{\min},\alpha_{\max}),$$

and the scaled image is represented as

(4) $$I_{s}=S\left(I_{c};\alpha \right),$$

where S(·) denotes the scale transformation operator. By continuously altering the relative size of small targets during training, more robust multi-scale representations are learned, effectively reducing the model’s dependence on fixed scale distributions. In addition to visual data, environmental sensor data, including temperature, humidity, and illumination, are synchronously collected, as these variables possess important ecological significance in pest occurrence and activity patterns. However, sensor data in real-world acquisition processes are often accompanied by noise, missing values, and inconsistent sampling frequencies, which can negatively affect vision–sensor collaborative modeling if directly fed into the model. Therefore, systematic cleaning and normalization of sensor data are required prior to data fusion. Let the sensor data vector collected at time *t* be denoted as s$\left(t\right)=[s_{1}\left(t\right),s_{2}\left(t\right),\ldots ,s_{K}\left(t\right)]$, where *K* represents the number of sensor variables. For outlier detection, a statistical distribution-based removal strategy is adopted. For each variable $s_{k}\left(t\right)$, its mean $\mu_{k}$ and standard deviation $\sigma_{k}$ are computed, and values outside a reasonable range are treated as anomalies, i.e.,

(5) $$|s_{k}\left(t\right)-\mu_{k}|>\lambda \sigma_{k},$$

where λ is an empirical threshold. After anomaly removal, missing values are filled using linear interpolation to ensure the continuity of the time series. Since the sampling frequencies of image data and sensor data are generally different, multimodal temporal alignment is further performed. Let the image acquisition timestamp be $t_{i}$ and the sensor sampling timestamp be $t_{s}$. Synchronization between image and sensor data is achieved by searching for the nearest neighbor time point that satisfies $|t_{i}-t_{s}|<\Delta t$, which can be formulated as

(6) $$s\left(t_{i}\right)=s(\arg\min_{t_{s}}\left|t_{i}-t_{s}\right|),$$

where Δt denotes the maximum allowable temporal deviation. This strategy ensures that each image is associated with a set of ecologically meaningful environmental variables, thereby providing reliable inputs for subsequent vision–sensor collaborative modeling. After cleaning and alignment, sensor data are further standardized to avoid training interference caused by differences in numerical scales among variables. Zero-mean unit-variance normalization is applied to each variable:

(7) $${\hat{s}}_{k}\left(t\right)=\frac{s_{k}\left(t\right)-\mu_{k}}{\sigma_{k}},$$

where ${\hat{s}}_{k}\left(t\right)$ denotes the normalized sensor variable. This process facilitates faster model convergence and improves the stability of multimodal feature fusion. Through the above procedures of image pre-processing, small-target enhancement, and sensor data cleaning and synchronization, a multimodal input system with spatial-scale and temporal consistency is constructed at the data level. This pre-processing and augmentation strategy not only enhances the perceptibility of small-target pests in the visual domain but also ensures the semantic and temporal alignment of environmental sensor information, thereby establishing a solid foundation for subsequent multi-scale visual feature modeling and environmental prior modulation modules and providing the necessary conditions for ecologically rational vision–sensor collaborative recognition.

### 3.3. Proposed Method

#### 3.3.1. Overall

After data processing and temporal alignment, a single input image is denoted as $X\in R^{H_{0}\times W_{0}\times C}$, and its corresponding environmental sensor vector is denoted as $s\in R^{K}$. The overall model follows a pipeline-style architecture consisting of multi-scale visual representation learning, environmental prior modulation, and vision–sensor collaborative discrimination. First, the image *X* is fed into the multi-scale visual feature modeling module, where a hierarchical backbone network extracts a feature pyramid $\left\{F_{1},F_{2},\ldots ,F_{L}\right\}$ from shallow to deep layers. The shallow feature $F_{1}$ preserves higher spatial resolution to capture texture and edge details of small targets, while the deep feature $F_{L}$ provides stronger semantic representations to suppress background interference. Subsequently, a cross-scale fusion unit aligns $\left\{F_{l}\right\}$ into a unified feature space and aggregates information to obtain an enhanced multi-scale visual representation $F^{ms}$, ensuring that small targets remain sufficiently distinguishable in deep semantic space. Next, the environmental sensor prior modulation module takes s as input and generates a modulation vector g through nonlinear mapping, which is applied to $F^{ms}$ for dynamic weighting and guidance, yielding the environment-modulated visual feature $\tilde{F}$. This process can be interpreted as an ecological-consistency-based recalibration of visual responses along channel or spatial dimensions, strengthening visual evidence under environment conditions consistent with pest occurrence while suppressing noise activations under mismatched conditions. Finally, the vision–sensor collaborative discrimination and classification module receives $\tilde{F}$ and constructs a joint discriminative representation *z*, which is processed by a lightweight classification head to perform feature compression and category separation, outputting pest category probabilities p=softmax(Wz+b). The representation *z* jointly encodes multi-scale fine-grained appearance cues and stable semantics constrained by environmental priors, enabling robust recognition of small-target pests such as aphids and thrips under complex backgrounds and varying conditions.

#### 3.3.2. Multi-Scale Small-Target Visual Feature Modeling Module

The multi-scale small-target visual feature modeling module takes the processed input image $X\in R^{H_{0}\times W_{0}\times C}$ as its starting point. The overall structure follows the design philosophy of multi-resolution parallel modeling, cross-scale relational fusion, and channel-adaptive enhancement, aiming to mitigate the progressive attenuation of small-target features in deep networks.

**Figure 2:**
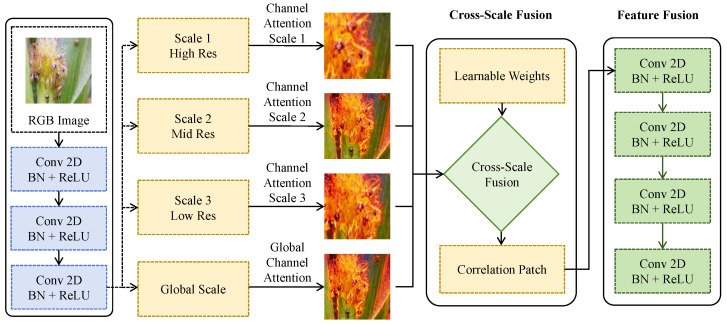
Illustration of the multi-scale small-target visual feature modeling module.

As shown in Figure 2, image features are first mapped layer by layer through a shared-parameter convolutional backbone network to obtain a set of hierarchical feature representations $\left\{F^{\left(1\right)},F^{\left(2\right)},F^{\left(3\right)},F^{\left(g\right)}\right\}$. Here, $F^{\left(1\right)}\in R^{H_{0}\times W_{0}\times C_{1}}$ represents high-resolution shallow features, $F^{\left(2\right)}$ and $F^{\left(3\right)}$ correspond to medium- and low-resolution features, respectively, and $F^{\left(g\right)}$ denotes a global-scale feature obtained via global receptive field modeling. Features at each scale are constructed using multiple 3×3 convolutional layers followed by batch normalization and nonlinear activation, ensuring consistent representational capacity across different spatial scales. To address the issue that small targets are easily overwhelmed in deep semantic layers, a channel attention mechanism is introduced at each scale to adaptively recalibrate the importance of feature channels. Specifically, global average pooling is applied to each scale feature $F^{\left(l\right)}$ to obtain a channel descriptor vector $z^{\left(l\right)}\in R^{C_{l}}$, which is then transformed through nonlinear mappings to generate channel weights $w^{\left(l\right)}=\sigma \left(W_{2}\delta \left(W_{1}z^{\left(l\right)}\right)\right)$, where δ(·) and σ(·) denote the activation function and normalization function, respectively. The weighted feature ${\hat{F}}^{\left(l\right)}=w^{\left(l\right)}⊙F^{\left(l\right)}$ explicitly enhances fine-grained channel responses associated with small targets, thereby reducing interference from background textures.

On this basis, cross-scale fusion is performed to enable information interaction among features at different resolutions. Specifically, features at each scale are first mapped to a unified spatial resolution via scale alignment operators, after which learnable weights $\left\{\alpha_{l}\right\}$ are introduced to combine features from different scales, yielding the fused feature

$$F^{ms}=\sum_{l}\alpha_{l}\;\cdot \;{\hat{F}}^{\left(l\right)},\;\sum_{l}\alpha_{l}=1.$$

This weighted fusion strategy avoids redundant representations caused by naive concatenation and allows the model to automatically balance high-resolution detail information and low-resolution semantic information according to task requirements. In addition, to enhance local consistency across scales, a cross-scale relational unit further models feature correlations within spatial neighborhoods, explicitly capturing correspondences of target regions across scales and improving continuous responses in densely distributed small-target areas. From a mathematical perspective, the introduction of multi-scale parallel mappings and channel-adaptive weighting in feature space is equivalent to imposing a set of scale-dependent reconstruction constraints on the original feature representation. As a result, feature responses corresponding to small targets are simultaneously amplified and kept consistent across multiple scales. This design effectively reduces discriminative instability caused by sparsity at a single scale, enabling small-target pests to maintain separable feature structures even in deep semantic space and providing a more robust and sufficient visual representation for subsequent environmental prior modulation and vision–sensor collaborative discrimination modules.

#### 3.3.3. Environmental Sensor Prior Modulation Module

The environmental sensor prior modulation module operates on the output of the multi-scale small-target visual feature modeling module. Its core objective is to introduce ecological constraints and perform learnable and interpretable dynamic modulation of visual features without altering the main discriminative structure of the visual backbone.

**Figure 3:**
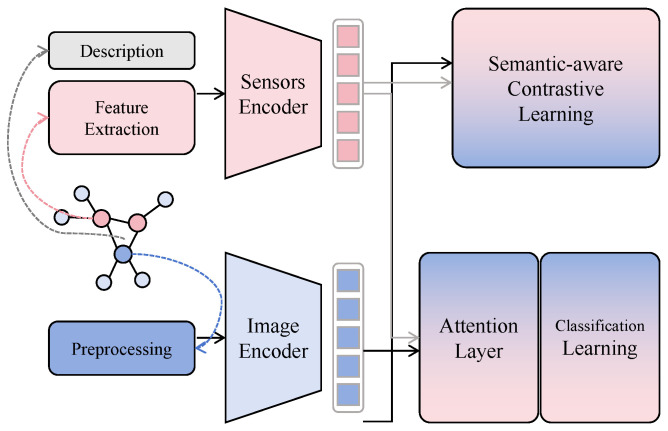
Illustration of the environmental sensor prior modulation module.

As shown in Figure 3, the fused multi-scale visual feature is denoted as $F^{ms}\in R^{H\times W\times C}$, where *H* and *W* represent spatial resolution and *C* denotes the number of channels. In this implementation, H=W=64 and C=256, indicating that the feature already integrates high-resolution details and low-resolution semantic information. Correspondingly, the environmental sensor input vector is denoted as $s\in R^{K}$ with K=3, representing air temperature, relative humidity, and illumination intensity, respectively, and is standardized to ensure comparable numerical scales. To map low-dimensional environmental variables to modulation signals applicable to high-dimensional visual features, a lightweight environmental encoding subnetwork is constructed. This subnetwork adopts a three-layer fully connected structure, with input dimension K=3, first hidden layer width $d_{1}=32$, second hidden layer width $d_{2}=128$, and output layer width equal to the visual feature channel number C=256. Through this design, environmental variables are progressively embedded into a modulation space aligned with visual channels. The mapping process is formulated as

(8) $$e_{1}=\phi \left(W_{1}s+b_{1}\right),\;e_{2}=\phi \left(W_{2}e_{1}+b_{2}\right),$$

(9) $$g=\sigma (W_{3}e_{2}+b_{3}),$$

where $W_{1}\in R^{32\times 3}$, $W_{2}\in R^{128\times 32}$, and $W_{3}\in R^{256\times 128}$. Here, ϕ(·) denotes a nonlinear activation function and σ(·) denotes a normalization mapping to stabilize the value range of the modulation vector $g\in R^{256}$. Each element of g can be interpreted as an importance weight for the corresponding visual channel under the current environmental condition.

The modulation vector g is then broadcast along spatial dimensions and applied to the visual feature $F^{ms}$ via channel-wise modulation, yielding the environment-constrained visual representation $\tilde{F}$:

(10) $${\tilde{F}}_{h,w,c}=g_{c}\cdot F_{h,w,c}^{ms},\;\forall h,w,c.$$

This operation is mathematically equivalent to introducing a diagonal modulation operator in feature space, which imposes environment-dependent scaling constraints on different semantic channels. Compared with direct concatenation of sensor features, this multiplicative modulation does not introduce additional spatial noise but instead reshapes the energy distribution of feature responses, systematically amplifying or suppressing visual activations in an ecologically consistent manner.

From a theoretical standpoint, if visual features are treated as random variables $F^{ms}$, their expected responses under different environmental conditions can be written as $E[F^{ms}|s]$. After introducing the modulation vector, the model effectively learns the conditional distribution $E\left[\tilde{F}|s\right]=E\left[g\left(s\right)⊙F^{ms}\right]$, which is equivalent, under a first-order approximation, to introducing environment-dependent prior weights in feature space, thereby reducing the variance of features inconsistent with environmental conditions. This property is particularly important for small-target pest recognition, where background noise often exhibits high instability across environments, while pest-related features tend to show stronger ecological consistency.

When combined with the multi-scale visual module, this modulation mechanism does not alter the spatial structure or scale relationships of $F^{ms}$ but operates along the channel dimension, reordering semantic responses of fused multi-scale features under environmental constraints. This serial design of visual modeling followed by prior modulation ensures that geometric and textural information of small targets is fully captured first, and then semantically constrained by environmental information, avoiding premature interference of environmental variables that could limit visual expressiveness. In the context of the task addressed in this study, the module significantly enhances discriminative channels for pest categories under high-risk environmental conditions such as high temperature and humidity, while suppressing spurious activations under unfavorable conditions, thereby improving recognition stability and generalization in complex agricultural scenarios while maintaining model simplicity and interpretability.

#### 3.3.4. Vision–Sensor Collaborative Discrimination and Classification Module

The vision–sensor collaborative discrimination and classification module takes the environment-modulated multi-scale visual representations as its primary input and uses sensor embeddings as conditional semantics to achieve consistent alignment and discriminative enhancement across scales.

**Figure 4:**
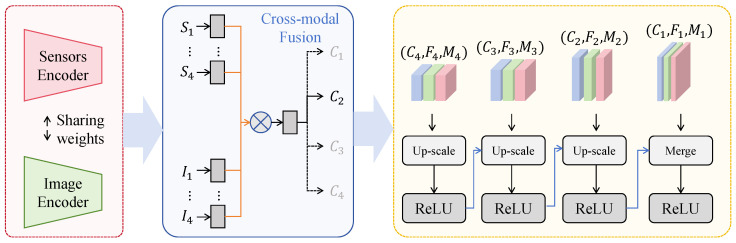
Illustration of the vision–sensor collaborative discrimination and classification module.

As shown in Figure 4, the four feature levels after multi-scale visual modeling and prior modulation are denoted as $\left\{{\tilde{F}}_{1},{\tilde{F}}_{2},{\tilde{F}}_{3},{\tilde{F}}_{4}\right\}$, with spatial sizes and channel numbers set as ${\tilde{F}}_{1}\in R^{64\times 64\times 64}$, ${\tilde{F}}_{2}\in R^{32\times 32\times 128}$, ${\tilde{F}}_{3}\in R^{16\times 16\times 192}$, and ${\tilde{F}}_{4}\in R^{8\times 8\times 256}$. The standardized sensor vector is denoted as $s\in R^{3}$. To provide controllable discriminative constraints across scales, a two-layer sensor encoder first maps s into a shared semantic space with dimension d=64:

(11) $$h=\psi \left(W_{a}s+b_{a}\right),\;u=\psi \left(W_{b}h+b_{b}\right),$$

where $W_{a}\in R^{32\times 3}$ and $W_{b}\in R^{64\times 32}$, and ψ(·) denotes a nonlinear activation function. The vector u is then projected through four scale-specific linear transformations to generate conditional vectors ${\left\{c_{i}\right\}}_{i=1}^{4}$:

(12) $$c_{i}=P_{i}u+q_{i},\;P_{i}\in R^{C_{i}\times 64},$$

where $\left(C_{1},C_{2},C_{3},C_{4}\right)=\left(64,128,192,256\right)$. For each scale, a conditional gating and residual injection mechanism is applied to fuse sensor semantics with local visual responses without disrupting multi-scale geometric structure. Specifically, a gating map is generated from $c_{i}$ and broadcast spatially, and the fused feature $M_{i}$ is obtained as

(13) $$G_{i}=\mathrm{reshape}\left(c_{i},1,1,C_{i}\right)⊗1_{H_{i}\times W_{i}},\;M_{i}={\tilde{F}}_{i}+\varphi \left({\tilde{F}}_{i}⊙G_{i}\right),$$

where $\left(H_{1},H_{2},H_{3},H_{4}\right)=\left(64,32,16,8\right)$, φ(·) denotes a 1×1 convolution followed by ReLU, and ⊗ indicates broadcasting. The fused features at all scales are then upsampled to 64×64 and aggregated:

(14) $${\bar{M}}_{1}=M_{1},\;{\bar{M}}_{2}=\mathrm{Up}\left(M_{2},2\right),\;{\bar{M}}_{3}=\mathrm{Up}\left(M_{3},4\right),\;{\bar{M}}_{4}=\mathrm{Up}\left(M_{4},8\right),$$

(15) $$F^{cs}=\rho \left({\mathrm{Conv}}_{1\times 1}\left[{\bar{M}}_{1}∥{\bar{M}}_{2}∥{\bar{M}}_{3}∥{\bar{M}}_{4}\right]\right),\;F^{cs}\in R^{64\times 64\times 256},$$

where Up(·) denotes bilinear upsampling, ∥ denotes channel concatenation, ${\mathrm{Conv}}_{1\times 1}$ compresses the concatenated 640 channels to 256, and ρ(·) denotes ReLU. The final classification head applies global average pooling followed by a two-layer perceptron to output probabilities for six pest categories. Let the pooled vector be $z\in R^{256}$:

(16) $$z=\mathrm{GAP}\left(F^{cs}\right),\;o=W_{2}\;\psi \left(W_{1}z+b_{1}\right)+b_{2},\;p=\mathrm{Softmax}\left(o\right),$$

where $W_{1}\in R^{128\times 256}$ and $W_{2}\in R^{6\times 128}$. The key advantage of this design lies in the fact that sensor information is not simply concatenated at the decision layer but participates in feature formation through scale-aligned conditional gating, enabling consistent reordering and stable activation of multi-scale visual responses under different environmental states. From a risk minimization perspective, let the true label be *Y*, visual features be *V*, and sensor variables be *S*. For any loss function *ℓ* and hypothesis class F, the optimal conditional predictor satisfies

(17) $$f^{★}=\arg\min_{f\in F}E\left[\ell (Y,f(V,S))\right],\;E\left[\ell (Y,f^{★}\left(V,S\right))\right]\leq E\left[\ell (Y,f^{★}\left(V,\bar{S}\right))\right],$$

where $\bar{S}$ denotes a degenerate constant condition independent of *V*. By conditioning visual representations on *S* and forming $M_{i}$ across scales, the realizable set of discriminative functions is effectively expanded, thereby reducing expected empirical risk under fixed model capacity and improving separability of small targets under complex backgrounds and scale variations.

## 4. Results and Discussion

### 4.1. Experimental Configuration

#### 4.1.1. Hardware and Software Platform

All experiments were conducted on a dedicated deep learning server equipped with an NVIDIA RTX 3090 GPU with 24 GB of memory, which was used to accelerate both model training and inference. The GPU provided sufficient parallel computing capability to support multi-scale feature extraction and batch-based processing during training. The server was further equipped with an Intel Xeon Gold 6226R CPU running at 2.90 GHz with 16 physical cores, along with 128 GB of system memory, ensuring efficient data loading, pre-processing, and caching of multimodal inputs. A high-speed solid-state drive (SSD) with a storage capacity of 2 TB was used to store raw image data, environmental sensor records, and intermediate experimental outputs, thereby minimizing input/output latency and improving overall training efficiency. This hardware configuration enabled stable execution of repeated experiments and cross-validation procedures on real-world agricultural datasets.

On the software side, all experiments were implemented under a Linux operating system (Ubuntu 20.04 LTS). Model development, training, and inference were carried out using the PyTorch deep learning framework (version 1.13.1), with CUDA (version 11.7) and cuDNN (version 8.5) providing GPU acceleration for numerical computation and tensor operations. Image pre-processing and data augmentation were performed using OpenCV (version 4.6.0) and the torchvision library (version 0.14.1), while environmental sensor data cleaning and normalization were implemented using NumPy (version 1.23.5) and SciPy (version 1.9.3). To ensure reproducibility, random seeds were fixed for the Python 3.10.12 environment, NumPy, and PyTorch throughout all experiments, and identical software configurations were maintained across all experimental runs.

During model training, the constructed dataset was divided into training, validation, and test sets, accounting for 70%, 15%, and 15% of the total data, respectively. To prevent potential spatial or temporal data leakage, samples collected from the same farmland plot and the same acquisition session were assigned to the same subset, ensuring strict independence across training, validation, and test partitions. This grouping strategy guarantees that no images captured from identical field locations or time periods appear in multiple subsets. Model optimization was performed using a gradient-based optimization algorithm, with the initial learning rate set to α and dynamically adjusted based on validation performance during training to mitigate overfitting. The batch size was set to *B* to balance GPU memory usage and training stability, and the number of training epochs was set to *E* until convergence was observed on the validation set. To further enhance the reliability and robustness of evaluation, a 5-fold cross-validation strategy was introduced on the training set. In each fold, non-overlapping grouped subsets were maintained, thereby reducing partition randomness while preserving spatial–temporal independence. The training set was partitioned into five non-overlapping subsets, with one subset selected as validation in each round and the remaining subsets used for training. The final performance was reported as the average over the five runs. This configuration effectively reduced the influence of data partition randomness and yielded more robust and reliable performance evaluation.

#### 4.1.2. Baseline Models and Evaluation Metrics

Multiple representative traditional methods and deep learning models were selected as baseline approaches to comprehensively evaluate the performance advantages of the proposed method in pest recognition tasks. The support vector machine model based on handcrafted features [49] constructs color, texture, and morphological descriptors and adopts the maximum margin classification criterion, thereby exhibiting stable discriminative capability under limited sample sizes. The random forest model [50] employs an ensemble learning mechanism based on multiple decision trees, enabling effective integration of diverse handcrafted features and improving adaptability to complex data distributions. Among deep learning baselines, the single-scale CNN [51] relies on an end-to-end feature learning framework to automatically extract discriminative representations from raw images, allowing effective characterization of local visual patterns of pests. ResNet [52] introduces residual connections to alleviate training difficulties in deep networks, thereby enhancing feature representation capacity while maintaining a deep architecture. Vision Transformer [53] adopts a self-attention mechanism to model global contextual relationships, which facilitates the capture of long-range semantic dependencies within images. The FPN-based multi-scale vision model [54] fuses features at different resolutions through a feature pyramid structure and demonstrates strong multi-scale modeling capability in small-target detection and recognition tasks. These baseline methods reflect the mainstream technical paradigms in current pest recognition research from different modeling perspectives and provide comprehensive and representative references for performance comparison with the proposed approach.

In the pest recognition experiments, multiple evaluation metrics were adopted to comprehensively assess both the overall classification performance and the recognition capability for small target pests. Accuracy was used to measure overall prediction correctness, Precision was employed to characterize the reliability of predictions for a specific pest category, Recall was used to reflect the detection capability for true pest samples, and the $F_{1}$ score was adopted to balance Precision and Recall, with particular emphasis placed on its performance for small-target pest categories. The mathematical definitions of the evaluation metrics are given as follows:

(18) $$Accuracy=\frac{TP+TN}{TP+TN+FP+FN},$$

(19) $$Precision=\frac{TP}{TP+FP},$$

(20) $$Recall=\frac{TP}{TP+FN},$$

(21) $$F_{1}=\frac{2\times Precision\times Recall}{Precision+Recall}.$$

Here, TP denotes the number of samples correctly identified as the target pest, TN denotes the number of samples correctly identified as non-target pests, FP denotes the number of samples incorrectly identified as the target pest, and FN denotes the number of pest samples that are present but not detected by the model. These variables are derived from the confusion matrix and constitute the fundamental components for evaluating classification model performance.

### 4.2. Overall Performance Comparison with Baseline Methods

This experiment was designed to systematically evaluate the pest recognition performance of the proposed method in real agricultural scenarios from an overall perspective and to compare it with multiple representative traditional approaches and deep learning baselines. To ensure statistical rigor, performance differences among all methods were analyzed using one-way analysis of variance (ANOVA). When a statistically significant overall effect was observed, Tukey’s honestly significant difference (HSD) post hoc test was conducted for pairwise comparisons. The significance level was set to p<0.05, and statistically significant differences are explicitly indicated in Table 2 using superscript symbols.

**Figure 5:**
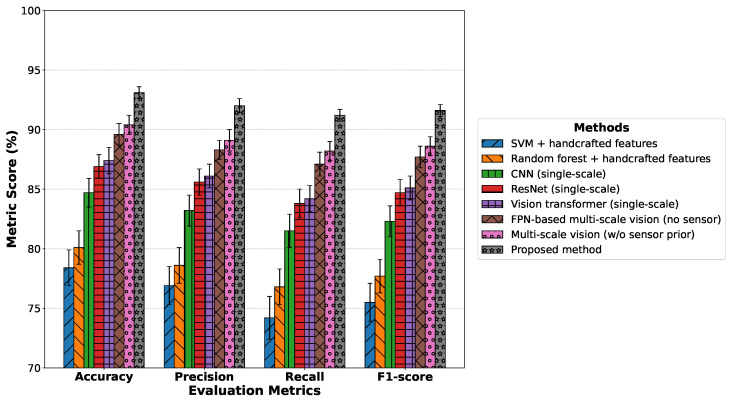
Overall performance comparison of different methods of pest recognition in terms of Accuracy, Precision, Recall, and F1 score.

As shown in Table 2 and Figure 5, the SVM and random forest methods based on handcrafted features achieve the lowest overall performance. Statistical analysis confirms that their results differ significantly from those of the proposed method (p<0.05). This limitation primarily arises from the fact that their feature spaces are constructed using fixed human-designed priors, which makes it difficult to characterize the high-dimensional nonlinear distributions exhibited by pests under complex backgrounds, especially in the presence of illumination variations, repetitive background textures, and extremely small-target scales, where decision boundaries between classes are prone to overlap. With the transition from traditional machine learning models to end-to-end deep learning approaches, the performance of single-scale CNN, ResNet, and Vision Transformer models gradually improves. Nevertheless, ANOVA results indicate that these improvements remain statistically inferior to the proposed multi-scale vision–sensor collaborative framework (p<0.05). After introducing multi-scale visual modeling, both the FPN-based method and the constructed multi-scale vision model achieve substantial improvements across all metrics. However, pairwise post hoc comparisons further demonstrate that incorporating environmental sensor priors yields statistically significant gains over purely vision-based multi-scale models. Consequently, the proposed method consistently achieves the highest Accuracy, Recall, and $F_{1}$ score, and the superiority over all baseline approaches is statistically validated under the defined significance criterion (p<0.05), thereby reinforcing both the empirical and statistical reliability of the results.

### 4.3. Recognition Performance Across Different Pest Categories

This experiment evaluates the model performance at the category level, aiming to analyze the stability and consistency of the proposed method across different pest types, and to examine whether the model exhibits significant bias toward specific sample distributions, morphological variations, or ecological habits. Unlike the overall performance comparison, this experiment emphasizes independent discriminative capability for each pest category rather than aggregated average results, thereby assessing whether the learned representations possess genuine generalization ability.

**Figure 6:**
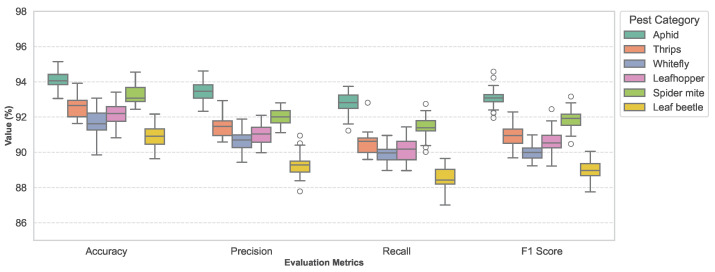
Performance distribution of different pest categories in terms of Accuracy, Precision, Recall, and F1 score.

As shown in Table 3 and Figure 6, the model achieves relatively balanced performance across all six pest categories. High-frequency, small-scale pests with relatively stable morphology, such as aphids and spider mites, exhibit the highest Accuracy and $F_{1}$ scores, indicating that the model can effectively exploit multi-scale visual features to capture their characteristic textures and shapes. For pests such as thrips, whiteflies, and leafhoppers, which are more prone to confusion with background textures and whose appearances vary significantly across growth stages, performance shows a moderate decrease but remains at a high level overall, demonstrating robust behavior under complex backgrounds. The leaf beetle category yields the lowest metrics, which is closely related to its smaller sample size, larger pose variation, and frequent local occlusions. Nevertheless, no severe performance imbalance is observed, suggesting that the model maintains a reasonable level of adaptability to long-tail categories.

It should be noted that the last row of this table reports macro-averaged results obtained by assigning equal weights to each pest category, whereas the corresponding results in Table 2 are computed by aggregating all test samples directly. Due to inherent imbalances in sample size and recognition difficulty across pest categories, macro-averaged metrics are more sensitive to rare or difficult classes and therefore tend to be lower than sample-level overall metrics. From a theoretical standpoint, performance differences across pest categories are fundamentally determined by their separability in feature space. Aphids and spider mites tend to form compact and stable clusters under multi-scale feature representations, resulting in clearer decision boundaries, whereas pests such as thrips, whiteflies, and leaf beetles exhibit higher similarity to leaf textures and background noise, leading to greater overlap in high-dimensional feature space. By enhancing multi-scale discriminability and imposing ecological constraints through environmental prior modulation, the proposed method suppresses feature responses inconsistent with environmental conditions and reduces inter-class performance disparity, demonstrating stable generalization across multiple small-target pest categories.

### 4.4. Ablation Study on Key Modules

The ablation study was designed to systematically verify the role and necessity of each key component in the proposed framework, and to analyze how different architectural choices affect the discriminative capability of the model, thereby ensuring that performance gains are not solely attributed to increased model complexity or parameter count.

**Figure 7:**
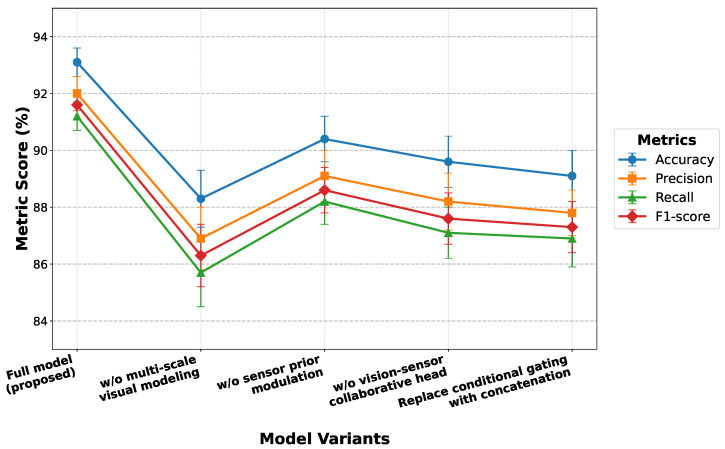
Performance trends of different model variants in the ablation study across Accuracy, Precision, Recall, and F1 score.

As shown in Table 4 and Figure 7, the complete model consistently achieves the best performance across all evaluation metrics, indicating strong complementarity among multi-scale visual modeling, environmental sensor prior modulation, and vision–sensor collaborative discrimination. Removing the multi-scale visual modeling module leads to the most pronounced performance degradation, demonstrating that single-scale representations are insufficient to capture the spatial complexity and scale variation of small pests, and that deep features alone lack adequate fine-grained expressiveness. In contrast, removing the sensor prior modulation or collaborative discrimination module results in relatively smaller but still substantial performance drops, highlighting that environmental information and cross-modal collaboration are not redundant but instead play a critical role in stabilizing decision boundaries in complex agricultural environments.

From a theoretical perspective, differences observed in the ablation results reflect fundamental changes in feature space construction. Without multi-scale modeling, the model performs discrimination within a single feature subspace, where feature distributions are more susceptible to overlap under scale variation and background interference, leading to unstable classification boundaries. The sensor prior modulation module constrains visual features through conditional environmental information, suppressing responses that are inconsistent with ecological conditions. Removing this module increases the degrees of freedom of the discriminative function in irrelevant feature directions, thereby introducing noisy activations. Replacing the vision–sensor collaborative structure with simple concatenation further reduces the model to linear multimodal fusion at the feature level, which lacks conditional alignment capability and prevents effective semantic coordination across modalities. Overall, the ablation study demonstrates that the proposed modules function synergistically to form an integrated framework, which is the fundamental reason why the complete model achieves superior performance in small-target pest recognition tasks.

### 4.5. Recognition Performance Under Crowded Colony Conditions

In practical agricultural environments, small-target pests frequently form dense colonies, where multiple individuals overlap within limited spatial regions. Such spatial aggregation and occlusion significantly increase intra-class texture redundancy and background confusion, posing additional challenges for recognition models. To explicitly evaluate model robustness under different colony densities, the original test set was further divided into two subsets according to manually annotated pest counts and spatial overlap levels: (1) a sparse subset, containing images with limited individual counts and weak spatial overlap; and (2) a dense subset, consisting of images characterized by severe aggregation and frequent occlusion. This partition was performed only on the test set and did not affect the training process. Four representative methods—including Vision Transformer (single-scale), FPN-based multi-scale vision (without sensor information), multi-scale vision without sensor prior modulation, and the proposed multi-scale vision–sensor collaborative framework—were evaluated separately on both subsets. The quantitative results are summarized in Table 5.

As shown in Table 5, all methods exhibit performance degradation under dense colony conditions compared to sparse scenarios. However, the magnitude of degradation differs substantially. Single-scale Vision Transformer shows a noticeable drop in both Accuracy and $F_{1}$ score, indicating sensitivity to severe overlap and texture crowding. Multi-scale visual modeling partially alleviates this issue by preserving high-resolution shallow features and cross-scale fusion, yet performance still declines when environmental priors are not incorporated. In contrast, the proposed method demonstrates significantly improved stability under dense conditions. Although a moderate performance decrease is observed compared to sparse samples, the degradation margin is smaller than that of all baseline methods. This improvement can be attributed to three factors: (1) multi-scale texture density modeling enhances discriminative representation of colony morphology; (2) cross-scale relational fusion captures spatial distribution structures across receptive fields; and (3) environmental prior modulation suppresses ecologically inconsistent background activations, thereby reducing noise amplification in crowded scenes. These results confirm that while the framework is designed for image-level recognition rather than instance-level segmentation, it maintains robust category discrimination capability even under severe aggregation and occlusion conditions.

### 4.6. Discussion

#### 4.6.1. Practical Implications for Intelligent Pest Monitoring

The proposed method demonstrates clear practical value in real agricultural production by effectively addressing the challenges of early-stage small-target pest detection under complex field conditions. In open-field farmland and greenhouse facilities in Bayannur City, Inner Mongolia, pests such as aphids, thrips, and whiteflies often emerge with low visual saliency and small population sizes, making timely manual inspection difficult. By integrating fixed visual monitoring with environmental sensors, the proposed multi-scale vision–sensor framework enables continuous and non-intrusive surveillance, allowing subtle visual signs of pest occurrence to be detected reliably. Moreover, the environmental prior modulation mechanism dynamically adjusts visual attention in response to changing temperature, humidity, and illumination conditions, reducing false alarms caused by background variation and short-term disturbances, and thereby improving the stability of pest recognition in real production environments.

Although the proposed framework demonstrates stable performance within the collected dataset, it is important to acknowledge that the current data are obtained from a single geographic region. Therefore, the generalization capability of the model across heterogeneous climate zones, different crop systems, and varying pest ecological dynamics requires further validation. Future research will incorporate multi-regional data collection and cross-region evaluation protocols to systematically assess transferability and improve model robustness.

#### 4.6.2. Implications for Agricultural Economics and Sustainable Management

Beyond technical performance, the proposed approach also exhibits important implications from an agricultural economics perspective. Accurate and early identification of pest occurrence provides a data-driven basis for precision pest management, enabling localized and timely control measures instead of broad, preventive pesticide application. This targeted intervention strategy can substantially reduce unnecessary pesticide input, lower chemical and labor costs, and mitigate negative environmental externalities, which collectively contribute to improved production efficiency and resource allocation in agricultural systems. In greenhouse production, early risk alerts allow growers to intervene before pest populations expand, reducing yield losses and avoiding costly large-scale treatments. In open-field agriculture, improved discrimination between true pests and background elements helps prevent unnecessary control actions, further reducing operational costs. By stabilizing crop yields and decreasing input variability, the proposed method supports income stability for farmers and enhances the economic resilience of regional agricultural production. From a long-term perspective, such intelligent monitoring technologies align with sustainable pest management strategies, facilitating the transition toward greener, more economically efficient plant protection practices.

### 4.7. Limitation and Future Work

Although the proposed multi-scale vision–sensor collaborative method achieves notable performance improvements in small-target pest recognition under complex agricultural scenarios, several aspects remain to be further explored. Crucially, from a biological standpoint, the current model leverages instantaneous weather and light conditions to correct recognition based on the acute behavioral responses of pests within a specific local microclimate. Consequently, the effect of this recognition correction will inevitably and radically change depending on the macro-weather conditions of different geographical areas and seasons. The environmental priors learned in this study are thus geographically and seasonally bounded. Furthermore, the current framework relies on coordinated data acquisition from fixed visual devices and environmental sensors, and its performance may be influenced by sensor deployment density and acquisition stability. Under extreme weather conditions or sensor occlusion, missing environmental information may affect the effectiveness of prior modulation. In addition, the proposed model focuses primarily on multi-scale feature modeling at the static image level, while pest migration, aggregation, and diffusion behaviors along the temporal dimension are not explicitly modeled. Pest population dynamics are fundamentally driven by cumulative and temporal climatic effects, such as temperature accumulation, long-term humidity fluctuations, and seasonal transitions, rather than just instantaneous conditions. The absence of explicit temporal environmental modeling constitutes a primary limitation of the present study. Future work will extend the proposed framework by incorporating time-series environmental data and continuous image sequences through temporal modeling architectures, such as Long Short-Term Memory networks or temporal Transformer models. This extension is expected to capture the dynamic evolution of pest occurrence and improve early warning timeliness. It should be noted that the environmental prior modeling in this study is based on instantaneous microclimate variables at the moment of image acquisition, without incorporating temporal-scale environmental dynamics. Although immediate temperature, humidity, and illumination conditions can reflect short-term behavioral responses of pests, diurnal variations, periodic climate fluctuations, and seasonal transitions may exert cumulative effects on pest population occurrence. Therefore, the absence of temporal modeling limits the framework’s ability to fully characterize ecological dynamics. Future work will integrate continuous time-series sensor data and temporal deep learning architectures to further enhance the ecological interpretability and long-term predictive capability of the multimodal collaborative framework.

It is also important to note that although the dataset spans April to November 2023 and covers major crops across multiple growth stages, the geographic scope remains concentrated within the Hetao Irrigation Area, a typical temperate arid and semi-arid agro-ecological zone characterized by large diurnal temperature variations and high summer temperatures with low precipitation. Pest behavioral responses and crop–environment interactions in this specific climatic context may differ substantially from those in the North China Plain, the humid middle–lower Yangtze River Basin, or southern protected agricultural systems. Therefore, the generalization capability of the current model under heterogeneous climate zones and different pest subspecies must be further validated. Future research will incorporate multi-site data collection across various climatic zones, combined with cross-regional validation and domain adaptation strategies to systematically evaluate the robustness and transferability of the proposed framework. In addition, integrating lightweight model design with edge computing techniques would facilitate deployment on field terminals, enabling low-latency online monitoring. Expanding the range of pest categories and exploring joint modeling with disease monitoring and crop growth assessment tasks would further contribute to the development of more comprehensive agricultural sensing systems. Additionally, acoustic sensing technologies have demonstrated considerable potential in detecting collective insect activity through the analysis of feeding sounds, wingbeat signals, and movement-induced vibrations [55]. By extracting temporal–frequency characteristics from acoustic signals, these approaches enable continuous monitoring of pest activity intensity and behavioral patterns under field conditions. In particular, spectral decomposition and energy distribution analysis allow for the identification of characteristic frequency bands associated with specific insect groups, thereby supporting early infestation detection and dynamic risk assessment [56]. Beyond purely acoustic approaches, distributed fiber-optic vibration monitoring systems have emerged as another promising sensing paradigm for large-scale agricultural environments [57]. Such systems leverage distributed acoustic sensing (DAS) principles to capture micro-vibration signals along extended fiber paths deployed across crop rows. Through spatiotemporal signal reconstruction, these technologies can achieve spatial localization of insect-induced disturbances, providing structural information about colony aggregation patterns and movement trajectories. Furthermore, vibration intensity profiling and spectral response modeling enable quantitative characterization of collective insect dynamics at the population level [58]. While these alternative sensing technologies offer valuable macro-level insights into pest activity distribution and colony-level behavior, they are typically optimized for activity detection, anomaly monitoring, or spatial localization rather than fine-grained species-level discrimination. In contrast, the proposed vision–sensor collaborative framework focuses on image-level morphological recognition under ecological constraints, explicitly modeling multi-scale visual texture representations and environmental priors to enhance discriminative robustness for small targets. Therefore, rather than functioning as competing methodologies, acoustic and distributed vibration sensing approaches can be regarded as complementary to visual perception systems. Acoustic and fiber-optic monitoring provide continuous, large-area structural awareness and dynamic colony information, whereas vision-based modeling enables precise categorical identification at the individual or image level. Future research will explore the integration of fiber-optic vibration signals, acoustic features, visual imagery, and environmental sensor data within a unified multimodal learning architecture, aiming to construct a spatially aware, hierarchically coordinated pest monitoring system that bridges colony-level dynamics and fine-grained visual recognition.

## 5. Conclusions

In complex agricultural production environments, achieving accurate and stable recognition of small-target pests constitutes a fundamental prerequisite for advancing precision plant protection management and green pest control. Owing to the small physical scale of pest individuals, strong background interference, and highly variable ecological conditions, traditional approaches based on handcrafted features or single-scale visual representations have difficulty achieving reliable performance in real-world scenarios. To address this challenge, a multi-scale vision–sensor collaborative pest recognition framework oriented toward real field and protected agriculture environments is constructed from the perspective of pest occurrence mechanisms and multimodal perceptual synergy, providing a new technical pathway for intelligent monitoring of small-target pests in complex scenes. The primary innovation of this study lies in the organic integration of multi-scale small-target visual modeling with environmental sensor priors, together with the realization of conditional representation learning through a vision–sensor collaborative discrimination mechanism. On the one hand, multi-scale visual feature modeling effectively alleviates the progressive attenuation of small-target representations in deep networks, thereby enhancing the perceptibility of fine-grained texture and morphological cues. On the other hand, environmental variables such as temperature, humidity, and illumination are introduced to modulate visual features as priors, enabling ecological constraints related to pest occurrence to be explicitly considered during the discrimination process and consequently improving recognition stability under complex backgrounds and varying environmental conditions. Experimental results demonstrate that the proposed method achieves approximately 93.1% Accuracy, 92.0% Precision, 91.2% Recall, and 91.6% $F_{1}$ score on the test set, significantly outperforming traditional machine learning approaches and multiple deep learning baseline models. Furthermore, category-level evaluations and ablation studies confirm the balanced performance of the model across different pest types as well as the effectiveness of each key module, indicating that the proposed approach not only exhibits superior overall performance but also maintains strong robustness in scenarios involving small sample sizes or high inter-class confusion.

## Figures and Tables

**Table 1:** Statistics of the multimodal pest dataset.

Data Type/Pest Category	Number of Samples	Acquisition Scenario	Acquisition Period
Aphid	3180	Field and greenhouse	2023.04–2023.11
Thrips	2460	Greenhouse	2023.04–2023.11
Whitefly	1985	Greenhouse	2023.05–2023.10
Leafhopper	1540	Field	2023.05–2023.09
Spider mite	1870	Field and greenhouse	2023.06–2023.10
Leaf beetle	1095	Field	2023.04–2023.08
Total pest image data	12,130	Field and greenhouse	2023.04–2023.11
Air temperature data	34,560	Field and greenhouse	2023.04–2023.11
Relative humidity data	34,560	Field and greenhouse	2023.04–2023.11
Illumination intensity data	25,920	Field and greenhouse	2023.05–2023.10

**Table 2:** Overall performance comparison of different methods on the test set (average results ± standard deviation of 5-fold cross-validation; statistical significance evaluated by one-way ANOVA followed by Tukey’s HSD post hoc test, significance level p<0.05).

Method	Accuracy (%)	Precision (%)	Recall (%)	$F_{1}$ (%)
SVM + handcrafted features	78.4 ± 1.5 *	76.9 ± 1.6 *	74.2 ± 1.8 *	75.5 ± 1.6 *
Random forest + handcrafted features	80.1 ± 1.4 *	78.6 ± 1.5 *	76.8 ± 1.5 *	77.7 ± 1.4 *
CNN (single-scale)	84.7 ± 1.2 *	83.2 ± 1.3 *	81.5 ± 1.4 *	82.3 ± 1.3 *
ResNet (single-scale)	86.9 ± 1.0 *	85.6 ± 1.1 *	83.8 ± 1.2 *	84.7 ± 1.1 *
Vision Transformer (single-scale)	87.4 ± 1.1 *	86.1 ± 1.0 *	84.2 ± 1.1 *	85.1 ± 1.0 *
FPN-based multi-scale vision (no sensor)	89.6 ± 0.9 *	88.3 ± 0.8 *	87.1 ± 1.0 *	87.7 ± 0.9 *
Multi-scale vision (w/o sensor prior)	90.4 ± 0.8 *	89.1 ± 0.9 *	88.2 ± 0.8 *	88.6 ± 0.8 *
Proposed method	93.1 ± 0.5	92.0 ± 0.6	91.2 ± 0.5	91.6 ± 0.5

* Indicates a statistically significant difference compared to the proposed method (p<0.05, one-way ANOVA).

**Table 3:** Classification performance of six pest categories (test set, average results of 5-fold cross-validation).

Pest Category	Accuracy (%)	Precision (%)	Recall (%)	$F_{1}$ (%)
Aphid	94.2	93.5	92.8	93.1
Thrips	92.6	91.3	90.5	90.9
Whitefly	91.8	90.6	89.7	90.1
Leafhopper	92.1	91.0	90.2	90.6
Spider mite	93.4	92.2	91.5	91.8
Leaf beetle	90.7	89.4	88.6	89.0
Macro-average	92.5	91.3	90.6	90.9

**Table 4:** Ablation study results of key modules (test set, average results ± standard deviation of 5-fold cross-validation; statistical significance assessed by one-way ANOVA followed by Tukey’s HSD post hoc test).

Variant	Accuracy (%)	Precision (%)	Recall (%)	$F_{1}$ (%)
Full model (proposed)	93.1 ± 0.5	92.0 ± 0.6	91.2 ± 0.5	91.6 ± 0.5
w/o multi-scale visual modeling	88.3 ± 1.0 *	86.9 ± 1.1 *	85.7 ± 1.2 *	86.3 ± 1.1 *
w/o sensor prior modulation	90.4 ± 0.8 *	89.1 ± 0.9 *	88.2 ± 0.8 *	88.6 ± 0.8 *
w/o vision–sensor collaborative head	89.6 ± 0.9 *	88.2 ± 1.0 *	87.1 ± 0.9 *	87.6 ± 0.9 *
Replace conditional gating with concatenation	89.1 ± 0.9 *	87.8 ± 0.8 *	86.9 ± 1.0 *	87.3 ± 0.9 *

* Indicates a statistically significant difference compared to the full model (p<0.05, one-way ANOVA).

**Table 5:** Recognition performance under different pest colony density conditions (sparse vs. dense subsets of the test set).

Method	Density	Accuracy (%)	Recall (%)	$F_{1}$ (%)
Vision Transformer (single-scale)	Sparse	88.6	85.9	86.8
Vision Transformer (single-scale)	Dense	84.9	81.7	83.1
FPN-based multi-scale (no sensor)	Sparse	90.8	88.3	89.2
FPN-based multi-scale (no sensor)	Dense	87.4	84.6	85.9
Multi-scale vision (w/o sensor prior)	Sparse	91.6	89.7	90.2
Multi-scale vision (w/o sensor prior)	Dense	88.8	86.1	87.3
Proposed method	Sparse	93.8	92.3	92.5
Proposed method	Dense	92.2	90.8	91.0

## Data Availability

The data and source code presented in this study are openly available in a public GitHub repository https://github.com/Aurelius-04/MVCF.git, accessed on 26 February 2026.
